# Intestinal dysbiosis is common in systemic sclerosis and associated with gastrointestinal and extraintestinal features of disease

**DOI:** 10.1186/s13075-016-1182-z

**Published:** 2016-11-29

**Authors:** Kristofer Andréasson, Zaid Alrawi, Anita Persson, Göran Jönsson, Jan Marsal

**Affiliations:** 1 Section of Rheumatology, Department of Clinical Sciences, Lund University, Lund, Sweden; 2 Section of Infectious Diseases, Department of Clinical Sciences, Lund University, Lund, Sweden; 3 Department of Clinical Sciences, Lund University, Lund, Sweden; 4 Immunology Section, Lund University, Lund, Sweden; 5 Department of Gastroenterology, Skåne University Hospital, Lund, Sweden

**Keywords:** Systemic sclerosis, Microbiome, Gastrointestinal, Dysbiosis

## Abstract

**Background:**

Recent evidence suggests a link between autoimmunity and the intestinal microbial composition in several rheumatic diseases including systemic sclerosis (SSc). The objective of this study was to investigate the prevalence of intestinal dysbiosis in SSc and to characterise patients suffering from this potentially immunomodulatory deviation.

**Methods:**

This study consisted of 98 consecutive patients subject to in-hospital care. Stool samples were analysed for intestinal microbiota composition using a validated genome-based microbiota test (GA-map™ Dysbiosis Test, Genetic Analysis, Oslo, Norway). Gut microbiota dysbiosis was found present as per this standardised test. Patients were examined regarding gastrointestinal and extraintestinal manifestations of SSc by clinical, laboratory, and radiological measures including esophageal cineradiography, the Malnutrition Universal Screening Tool (MUST), levels of plasma transthyretin (a marker of malnutrition) and faecal (F-) calprotectin (a marker of intestinal inflammation).

**Results:**

A majority (75.5%) of the patients exhibited dysbiosis. Dysbiosis was more severe (r_s_ = 0.31, *p* = 0.001) and more common (*p* = 0.013) in patients with esophageal dysmotility. Dysbiosis was also more pronounced in patients with abnormal plasma levels of transthyretin (*p* = 0.045) or micronutrient deficiency (*p* = 0.009). In 19 patients at risk for malnutrition according to the MUST, 18 exhibited dysbiosis. Conversely, of the 24 patients with a negative dysbiosis test, only one was at risk for malnutrition. The mean ± SEM levels of F-calprotectin were 112 ± 14 and 45 ± 8 μg/g in patients with a positive and negative dysbiosis test, respectively. Dysbiosis was more severe in patients with skin telangiectasias (*p* = 0.020), pitting scars (*p* = 0.023), pulmonary fibrosis (*p* = 0.009), and elevated serum markers of inflammation (*p* < 0.001). However, dysbiosis did not correlate with age, disease duration, disease subtype, or extent of skin fibrosis.

**Conclusions:**

In this cross-sectional study, intestinal dysbiosis was common in patients with SSc and was associated with gastrointestinal dysfunction, malnutrition and with some inflammatory, fibrotic and vascular extraintestinal features of SSc. Further studies are needed to elucidate the potential causal relationship of intestinal microbe-host interaction in this autoimmune disease.

## Background

Systemic sclerosis (SSc) is an autoimmune systemic disease of unknown etiology. Genetic factors may only partly explain the pathobiology, and as yet uncharacterised environmental factors have been suggested to have a major influence on the development of SSc [1]. The number of bacteria in the human gastrointestinal (GI) tract has been estimated to 10^14^, reaching a biomass of around 2 kg [2]. In both health and disease, these microbiota are in continuous interaction with the epithelium and immune cells of the GI mucosa, and have profound effects on the host’s local and systemic immune system [3]. Maintenance of a balanced bidirectional interaction has been suggested to be essential in preventing development and progression of autoimmune diseases [4].

Altered microbiota composition, commonly referred to as dysbiosis, has been shown to induce and modulate systemic inflammation in animal models of rheumatic diseases and other immune-mediated inflammatory diseases (IMIDs) [5–7]. In the field of rheumatology, intestinal dysbiosis has been associated with rheumatoid arthritis (RA), systemic lupus erythematosus, Sjögren’s syndrome and ankylosing spondylitis [7–11]. A randomised double-blind placebo-controlled clinical trial in RA patients indicated that disease activity may be sensitive to modulation of gut microbiota through ingestion of probiotics [12]. In contrast, a similar trial did not show any significant differences between probiotics and placebo [13].

In SSc, small intestinal bacterial overgrowth is a well-described complication associated with GI dysmotility, GI discomfort, and malnutrition [14, 15]. Successful treatment of small intestinal bacterial overgrowth in SSc leads to improvement in GI symptoms [14]. Recently, alterations also in the colonic microbial composition in SSc have been reported [16].

Assessment of GI disease in SSc is challenging. Esophageal cineradiography has been suggested as the gold standard in the objective assessment of GI SSc [17]. Others and we have suggested that faecal calprotectin (F-calprotectin) constitutes a feasible tool in the evaluation of GI SSc [15, 18]. Malnutrition is one facet of GI disease that has been linked not only to morbidity and decreased quality of life, but also to increased mortality [19]. The Malnutrition Universal Screening Tool (MUST) is a validated method for identifying SSc patients at risk for malnutrition [20]. Decreased plasma levels of transthyretin, also known as prealbumin, represent a biomarker of malnutrition that also predicts mortality in SSc [19, 21].

The objective of this study was to examine the prevalence of dysbiosis in SSc. Furthermore, we aimed at exploring how intestinal dysbiosis relates to extraintestinal as well as gastrointestinal manifestations of SSc, including malnutrition.

## Methods

### Patients

Consecutive patients fulfilling the American Congress of Rheumatology/European League Against Rheumatism (ACR/EULAR) 2013 classification criteria for SSc and subject to planned in-hospital care due to SSc at the Skane University Hospital in Lund, Sweden between April 2014 and October 2015, were invited to this study. Out of 226 patients, 100 subjects both agreed to participate and were able to provide a fresh stool sample during their in-hospital stay. Patients with inflammatory bowel disease (IBD), intestinal malignancy, and/or colostomy were excluded (n = 2). In total, the study cohort consisted of 98 patients.

### Ethics

The study was approved by the Regional Ethics Review Board, Lund, Sweden, reference number 2011/596. Informed written consent was obtained from all patients before study inclusion and the study conformed to the ethical guidelines of the Declaration of Helsinki.

### Clinical assessment

The following data were collected: age, sex, and disease duration (defined both as years since onset of Raynaud’s phenomenon [RP] and years since the first non-RP manifestation). Patients were classified as having either diffuse cutaneous SSc (dcSSc) or limited cutaneous SSc (lcSSc) [22]. Esophageal function was assessed by cineradiography and evaluated by a radiologist, as previously described [23]. The cineradiograms were obtained by recording the swallowing of barium contrast in upright and prone positions using a high-speed camera. Esophageal motility dysfunction was categorised as absent, mild, moderate, or severe. Skin involvement was assessed using the modified Rodnan skin score (mRSS) [24]. The presence or absence of skin telangiectasia and pitting scars were noted. Pulmonary function was evaluated using a body plethysmograph (Erich Jaeger GmbH, Hoechberg, Germany). Lung fibrosis was identified by high-resolution computed tomography. Echocardiography was performed on all patients, and pulmonary arterial hypertension (PAH) was diagnosed by means of right heart catheterisation.

### Assessment of medical records

Medical records were systematically studied. Height and weight were noted as well as weight change during the last 12 months. Individual MUST scores were calculated as previously described [20]. A MUST score of 0 represents low risk for malnutrition, a score of 1 medium risk, and a score of ≥2 high risk. Patients’ usage of prescribed drugs including proton pump inhibitors (PPIs), antibiotics, glucocorticoids and immunosuppressive agents were noted.

### Assessment of intestinal symptoms

All patients were systematically questioned regarding the following GI symptoms: heartburn (dyspepsia), dysphagia, diarrhea, and/or constipation. These were recorded as present or not.

### Laboratory examinations

Blood tests included measurements of C-reactive protein (CRP), erythrocyte sedimentation rate (ESR), haptoglobin, orosomucoid, α_1_-antitrypsin, immunoglobulin (Ig)G, IgM, IgA, vitamin B_12_, folic acid, ferritin, iron, transferrin iron-binding capacity (TIBC) and transthyretin. Subjects with an iron/TIBC ratio < 0.16 were considered to be iron deficient [25]. F-calprotectin was measured using a commercially available enzyme-linked immunosorbent assay (ELISA, Calpro, Lysaker, Norway). The lower limit of the ELISA was 30 μg/g and values below this cutoff were estimated as 20 μg/g. In accordance with published data and recommendations from the manufacturer, we considered F-calprotectin levels < 50 μg/g to be within normal range [26].

### Assessment of gut dysbiosis

The GA-map™ Dysbiosis Test (Genetic Analysis, Oslo, Norway) has been developed and validated in relation to a Scandinavian control population to identify dysbiosis in adults by genetic analysis of a stool sample. The test makes use of 54 bacterial ribosomal RNA probes specific for various intestinal bacterial species or clades to generate genomic data on the intestinal microbiota composition. Using a defined algorithm, these data are subsequently translated into a Dysbiosis Index Score ranging from 1 to 5 (grades 1–2 are defined as eubiosis and 3–5 as dysbiosis). The test has been compared with MiSeq Illumina sequencing-based protocols and proven successful in identifying dysbiosis [6, 27]. In a healthy control population, 84% exhibited eubiosis and 16% dysbiosis [27]. In the current study, gut microbiota eubiosis and dysbiosis were delineated as per the standardised GA-map™ Dysbiosis Test results.

### Statistical analyses

The Mann-Whitney *U* test was used to compare the degree of dysbiosis and the χ2 test to compare the frequency of dysbiosis in patients with and without various manifestations of SSc. Spearman correlation coefficient (r_s_) was used to correlate the Dysbiosis Index Score with other continuous variables.

## Results

### Study population characteristics and levels of dysbiosis

**Fig. 1:**
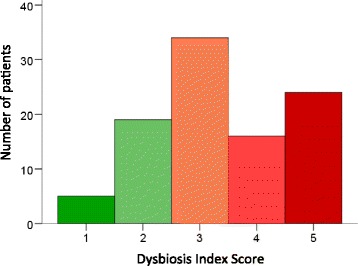
Dysbiosis is common in patients with systemic sclerosis.A majority of the study population suffers from dysbiosis, as defined by the GA-map™ Dysbiosis Test, with 25% exhibiting pronounced dysbiosis

Systemic sclerosis patients (n = 98) were examined for an array of characteristics and assessed for intestinal dysbiosis analysing their stools using the GA-map™ Dysbiosis Test. Patient characteristics are presented in Table 1. A majority (75.5%) of the patients exhibited dysbiosis to some degree (score 3–5), and a significant proportion (24.9%) suffered from severe dysbiosis (score 5, Fig. 1).

**Table 1:** Patient characteristics

	n	(%)
Systemic sclerosis subtype		
*limited cutaneous SSc*	77	(78)
*diffuse cutaneous SSc*	21	(22)
Autoantibodies		
*ANA*-*positive*	87	(89)
*ACA*-*positive*	33	(34)
*ARA*-*positive*	10	(10)
*ATA*-*positive*	11	(11)
Smoking		
*smoker*	11	(11)
*ex*-*smoker*	43	(44)
*non*-*smoker*	44	(45)
Telangiectasias	39	(40)
Pulmonary arterial hypertension^a^	13	(13)
Pitting scars, current	23	(23)
Lung fibrosis^b^	35	(36)
Pathological cineradiography	82	(84)
Regular PPI usage	78	(80)
Immunosuppressive therapy		
*mycophenolate mofetil*	23	(23)
*methotrexate*	5	(5)
*azathioprine*	10	(10)
*no immunosuppressive therapy*	60	(61)
	median	interquartile range
Modified Rodnan skin score		
*limited cutaneous*	2	(0, 4)
*diffuse cutaneous*	10	(4, 22)
Disease duration, years^c^	6	(2, 16)
Prednisolone, daily intake (mg)	0	(0, 4)

*ANA* anti-nuclear antibodies, *ACA* anti-centrome antibodies, *ARA* anti-RNA polymerase III antibodies, *ATA* anti-topoisomerase1 antibodies, *PPI* proton pump inhibitor

^a^As determined by right heart catheterisation

^b^As determined on high-resolution computed tomography

^c^Years since first non-Raynaud’s phenomena symptom

### Dysbiosis was associated with gastrointestinal manifestations of systemic sclerosis

**Fig. 2:**
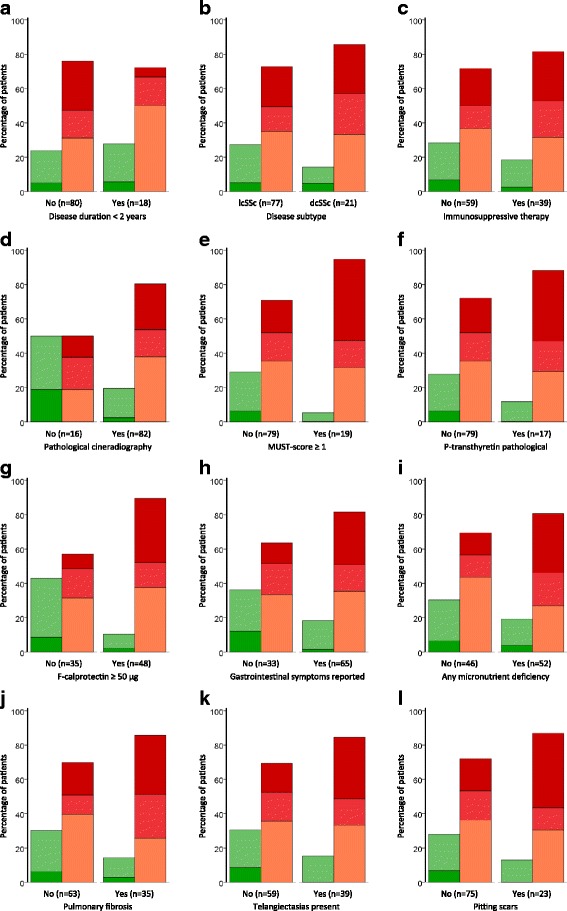
Dysbiosis correlates with gastrointestinal and some extraintestinal manifestations of SSc, but not disease subtype or immunosuppressive therapy. Dysbiosis was prevalent in patients with both short and long disease duration (**a**), lcSSc and dcSSc (**b**) as well as in patients with and without immunosuppressive therapy (**c**), with no significant differences between groups. Dysbiosis was more pronounced in patients with gastrointestinal manifestations of SSc including pathological oesophageal function, *p* = 0.036 (**d**); at risk for malnutrition, *p* = 0.005 (**e**); low levels of P-transthyretin, *p* = 0.045 (**f**); increased levels of F-calprotectin, *p* < 0.001 (**g**); gastrointestinal symptoms present, *p* = 0.019 (**h**) or micronutrient deficiency *p* = 0.009 (**i**). Also, patients with pulmonary fibrosis, *p* = 0.009 (**j**); telangiectasias, *p* = 0.020 (**k**); or pitting scars, *p* = 0.023 (**l**) had more pronounced dysbiosis compared to other patients. *dcSSc* diffuse cutaneous SSc, *F-calprotectin* faecal calprotectin, *lcSSc* limited cutaneous SSc, *MUST* Malnutrition Universal Screening Tool

A majority of the patients (84%) exhibited esophageal dysfunction, and dysbiosis was significantly more common in this group (*p* = 0.013; Fig. 2). The degree of dysmotility correlated with intestinal dysbiosis (Table 2). Malnutrition was frequent; 53% of the patients exhibited deficiency of folic acid, vitamin B12, and/or iron. Nineteen patients had a MUST score of ≥ 1, of which 18 exhibited dysbiosis, and 17 patients had pathological levels of P-transthyretin of which 15 exhibited dysbiosis. Patients with these malnutrition-associated characteristics (any deficiency, MUST ≥ 1, and/or abnormal transthyretin levels) displayed a higher degree of dysbiosis (Fig. 2) compared to the other subjects. Similarly, patients with any self-reported GI symptoms (Fig. 2) and patients using PPIs had a higher degree of dysbiosis compared to the other subjects (*p* = 0.019 and *p* = 0.002, respectively). Subanalysis of different types of self-reported GI symptoms did not reveal any significant associations. A majority of the SSc subjects exhibited abnormal F-calprotectin levels which were associated with the degree of dysbiosis (Fig. 2, Table 2). The mean ± SEM levels of F-calprotectin were 112 ± 14 and 45 ± 8 μg/g in patients with a positive and negative dysbiosis test, respectively.

**Table 2:** Correlation between the Dysbiosis Index Score and laboratory markers of inflammation, and disease characteristics, respectively

	n	Spearman’s correlation coefficient (r_s_)	*p* value
*Laboratory markers of inflammation*			
C-reactive protein	98	0.35	<0.001
Haptoglobin	98	0.34	<0.001
Orosomucoid	98	0.39	<0.001
α_1_-antitrypsin	98	0.27	0.007
Erythrocyte sedimentation rate	98	0.16	0.156
IgA	98	0.13	0.266
IgM	98	−0.05	0.654
IgG	98	−0.05	0.632
Faecal calprotectin	83	0.38	<0.001
*Disease characteristics*			
Years since onset of RP	94	−0.07	0.501
Years since the first non-RP symptom	89	0.09	0.383
Patient’s age at dysbiosis analysis	98	0.08	0.413
modified Rodnan skin score	98	0.05	0.659
Dysmotility of oesophagus	97	0.31	0.002

*Ig* immunoglobulin, *RP* Raynaud’s phenomenon

### Dysbiosis was associated with certain extraintestinal manifestations of systemic sclerosis

The degree of dysbiosis was analysed in reference to major fibrotic and vascular extraintestinal manifestations of SSc. Dysbiosis frequencies and severity did not differ between patients with dcSSc and lcSSc (Fig. 2), and the degree of dysbiosis did not correlate with the extent of skin disease (Table 2). However, dysbiosis was more pronounced among patients with pulmonary fibrosis (Fig. 2). We were unable to identify any association between the degree of dysbiosis and vital capacity or carbon monoxide diffusing capacity, (r_s_ = -0.126, *p* = 0.216, n = 98; r_s_ = -0.172, *p* = 0.232, n = 96). Among the 98 patients, 13 (13.3%) suffered from PAH. Dysbiosis was not more common or more severe among these (*p* = 0.316). However, dysbiosis was more severe among the 39 patients exhibiting skin telangiectasia, and among the 23 patients with pitting scars (Fig. 2). Dysbiosis was not more severe or prevalent among subjects with antibodies against centromere, topoisomerase 1, or RNA polymerase III. The degree of dysbiosis did not correlate with usage of glucocorticoids (r_s_ = 0.15, *p* = 0.139) and was not associated with usage of immunosuppressive therapy or antibiotics (*p* = 0.344 and *p* = 0.684, respectively).

### Dysbiosis was associated with laboratory markers of inflammation

Routine blood tests addressing systemic inflammation were assessed and correlated with the degree of dysbiosis. The grade of dysbiosis correlated with levels of CRP, haptoglobin, orosomucoid, and α_1_-antitrypsin, but not with the levels of ESR, IgG, IgM or IgA (Table 2). Of note, all three patients with IgA levels above reference levels had a Dysbiosis Index Score of 5 (*p* = 0.059).

### Dysbiosis was common also in patients with early systemic sclerosis

Disease duration was defined by two different measures and subsequently correlated with the degree of dysbiosis. The Dysbiosis Index Score did not correlate either with disease duration defined as years since RP debut or disease duration defined as years since first non-RP symptom or age (Table 2). Dysbiosis was prevalent among patients with less than 2 years since the debut of RP or first non-RP symptom (73% and 72%, respectively), similarly to patients with more long-standing disease (76% and 76%, Fig. 2).

### Analysis of specific bacterial genera

In a secondary analysis, we examined the frequency of specific bacterial genera and species previously associated with SSc, included in the GA-map™ Dysbiosis Test. A large proportion of patients with SSc exhibited low levels of *Faecalibacterium prausnitzii* (66/98; 67.3%) and/or *Clostridiaceae* (25/98; 25.5%) compared to eubiotic individuals. Also, relatively high levels of *Lactobacillus* (31/98; 31.6%) but not *Bifidobacterium* (6/98; 6.1%) were common among our subjects.

## Discussion

In this cross-sectional study encompassing 98 SSc patients we show that intestinal dysbiosis is common in SSc and is related to GI manifestations of disease. Also, we show that dysbiosis is associated with certain extraintestinal SSc features of inflammatory, vascular, and fibrotic type. We present data showing that intestinal dysbiosis is already present early in the course of SSc, indicating that dysbiosis may precede initial signs of fibrosis.

Several IMIDs have been associated with alterations in the microbial composition in the intestine, including RA, systemic lupus erythematosus, Sjögren’s syndrome, and IBD [7, 8, 11, 28]. Among human IMIDs, dysbiosis has been most extensively studied in IBD. These patients display decreased diversity in their gut microbiota, increased numbers of bacteria driving inflammatory activity, and decreased numbers of bacteria with immunoregulatory effects [28]. An important question is whether IBD-associated dysbiosis is a primary or secondary phenomenon. In animal models of IBD both loss of immunoregulatory and addition of disease-promoting bacteria have been shown to contribute to disease activity, supporting a primary disease-driving role for dysbiosis [29]. In IBD patients, various strategies for manipulating the gut microbiota, including exclusive enteral nutrition, prebiotics, probiotics, postbiotics, and faecal microbiota transplantation have shown mixed but overall promising results [30].

Molecular analyses have revealed some similarities between the process of IBD-associated intestinal fibrosis and SSc-associated skin fibrosis, including transforming growth factor beta (TGF-β) and peroxisome proliferator-activated receptor-dependent pathways resulting in collagen I production by fibrocytes and fibroblasts [31, 32]. Furthermore, while inflammation can be treated by immunosuppressive therapy, these fibrotic processes are resilient also to modern therapy in both diseases. In IBD as well as SSc, elevated F-calprotectin levels are common, indicating intestinal inflammation. Similar to data presented in this study, increased F-calprotectin levels have been associated with dysbiosis also in IBD [33].

Volkmann et al. recently reported altered microbial colonic mucosal composition in 17 SSc patients [16]. Our study comprising 98 SSc patients corroborates this finding, as we show a high incidence of dysbiosis in our patients. We also report low levels of the immunoregulatory bacteria *Faecalibacterium prausnitzii,* which is in agreement with studies in IBD [34]. In accordance with Volkmann et al.*,* we report high levels of *Lactobacillus* in SSc patients, which contrasts this disease from several other IMIDs [4]. As previously suggested, this finding might raise novel questions regarding the usage of *Lactobacilli-*containing probiotics in SSc.

Our report is based on faecal analyses and not analyses on colonic lavage or intestinal biopsies. Consequently, a weakness of our approach is the inability to specifically focus on bacteria prevalent in the interface between the colonic mucosa and the intestinal lumen. It is noteworthy that even though different methodologies were used, our major finding is consistent with Volkmann’s report.

Objective evaluation of GI disease in SSc is challenging. SSc can affect the GI tract in several different ways including dysmotility, malnutrition, inflammation, and fibrosis. In our study, we evaluated the GI tract by assessment of esophagus motility using barium cineradiography which has previously been suggested as the gold standard in objective evaluation of this disease [17]. We investigated malnutrition by laboratory markers including P-transthyretin, and anthropometric data using the MUST [19, 20]. While malnutrition in SSc has been suggested to be caused by malabsorption [35], additional mechanisms are likely to be involved including cachexia caused by the chronic inflammatory process [36].

A majority of our patients were prescribed PPI and usage of this medication was interestingly enough associated with dysbiosis. However, previous studies have failed to show that PPI usage per se causes significant aberrations in colonic microbiota composition [37]. In agreement with a previous study and interpretation by Krause et al. [36], we suggest that regular use of PPI primarily is an unspecific marker of symptomatic GI SSc. In this study, all patients were questioned about GI symptoms, and indeed, dysbiosis was more common in patients with GI symptoms indicating that a validated questionnaire, such as the UCLA SCTC GIT 2.0 should be included in future studies [38].

The primary aim of this study was to study the prevalence of dysbiosis in SSc. Unlike whole-genome sequencing studies, we have only limited data on specific bacterial genera. Furthermore, we do not have data on intestinal metabolic pathways used by the different microbiomes our patients harbor. Further studies encompassing such analyses are needed to further elucidate the intricate relationship between the host and the microbiome in SSc [39].

We can only speculate on the mechanisms behind the associations between dysbiosis and GI or extraintestinal manifestations of SSc. It can be hypothesised that several of these manifestations are indirect markers of severe disease. However, we were unable to identify an association between the mRSS, disease subtype, autoantibody profile, and immunosuppressive therapy. Taking this into consideration, we are therefore inclined to suggest that the relationship between the intestinal microbiome and SSc is multifactorial and related to factors independent of disease severity or autoantibody status. We note that dysbiosis is associated with increased serum levels of markers of inflammation and we suggest that further studies are warranted to elucidate the impact of GI dysbiosis on the immune system in SSc.

## Conclusions

Examining a large cross-sectional cohort of SSc patients we report that intestinal dysbiosis is prevalent in early as well as late disease, and associated with both GI and extraintestinal manifestations of SSc. Given our current knowledge from other IMIDs, we suggest that an aberration of the intestinal microbiota may contribute to the development of systemic inflammation and fibrosis, although causal relationships remain to be established.

## Abbreviations

ACA: anti-centromeric antibodies
ACR/EULAR: American Congress of Rheumatology/European League Against Rheumatism
ANA: anti-nuclear antibodies
ARA: anti-RNA-polymerase III antibodies
ATA: anti-topoisomerase antibodies
CRP: C-reactive protein
dcSSc: diffuse cutaneous SSc
ELISA: enzyme-linked immunosorbent assay
ESR: erythrocyte sedimentation rate
F-calprotectin: faecal calprotectin
GI: gastrointestinal
IBD: inflammatory bowel disease
Ig: immunoglobulin
IMID: immune-mediated inflammatory disease
lcSSc: limited cutaneous SSc
mRSS: modified Rodnan skin score
MUST: Malnutrition Universal Screening Tool
PAH: pulmonary arterial hypertension
PPI: proton pump inhibitor
RA: rheumatoid arthritis
RP: Raynaud’s phenomenon
SSc: systemic sclerosis
TGF-β: transforming growth factor beta
TIBC: total iron-binding capacity
